# Malaria, helminths and malnutrition: a cross-sectional survey of school children in the South-Tongu district of Ghana

**DOI:** 10.1186/s13104-016-2025-3

**Published:** 2016-04-27

**Authors:** Patrick Ferdinand Ayeh-Kumi, Kantanka Addo-Osafo, Simon Kwaku Attah, Patience Borkor Tetteh-Quarcoo, Noah Obeng-Nkrumah, Georgina Awuah-Mensah, Harriet Naa Afia Abbey, Akua Forson, Momodou Cham, Listowell Asare, Kwabena Obeng Duedu, Richard Harry Asmah

**Affiliations:** Department of Medical Laboratory Sciences, School of Biomedical and Allied Health Sciences, University of Ghana, Korle-bu, Accra, Ghana; Department of Microbiology, University of Ghana School of Biomedical and Allied Health Sciences, Accra, Ghana; Department of Medical Laboratory Sciences, University of Ghana School of Biomedical and Allied Health Sciences, Accra, Ghana; Comboni Catholic Hospital, Sogakope, Ghana; Department of Biomedical Sciences, School of Basic and Biomedical Sciences, University of Health & Allied Sciences, Ho, Ghana

**Keywords:** Malaria, Haemoglobin, Schistosomiasis, Malnutrition, Stunting, School, Children, Ghana

## Abstract

**Background:**

As part of malaria characterization study in the South-Tongu district of Ghana, the current study was conducted to explore relationships between malaria, schistosomiasis, soil transmitted helminths and malnutrition in riparian community settings that had hitherto encountered episodes of mass deworming exercises.

**Methods:**

School-age children were enrolled in a cross-sectional study from April through July 2012. Stool and urine samples were examined respectively for helminths and *Schistosoma haematobium*. Blood samples were analyzed for malaria parasites and haemoglobin (Hb) concentrations, respectively. Anthropometric indices were measured. Relationships were determined using generalized linear models.

**Results:**

The results show low numbers of asymptomatic *Plasmodium falciparum* (9.2 %, n = 37/404) and *S. haematobium* (2.5 %, n = 10/404) infections. The associations between significance terms in the multivariate analysis for *P. falciparum* infections were further assessed to test the significance of the product terms directly i.e., age in years [adjusted odds ratio (AOR), 3.1; 95 % confidence interval (CI) 1.1–5.6], Hb concentration (AOR = 0.71; 95 % CI 0.42–2.3), and stunted malnutrition (AOR, 8.72; 95 % CI 4.8–25.1). The *P. falciparum*-associated decrease in mean Hb concentration was 2.82 g/dl (95 % CI 1.63–4.1 g/dl; *P* = 0.001) in stunted children, and 0.75 g/dl (95 % CI 1.59–0.085 g/dl; *P* = 0.076) in the non-stunted cohort. The anaemia-associated decrease in mean parasitaemia in stunted children was 3500 parasites/µl of blood (95 % CI 262.46–6737.54 parasites/µl of blood; *P* = 0.036), and in non-stunted children 2127 parasites/µl of blood (95 % CI −0.27 to 4.53; *P* = 0.085). Stunted malnutrition was the strongest predictor of *S. haematobium* infection (AOR = 11; 95 % CI 3.1–33.6) but significant associations as described for *P. falciparum* infections were absent. The population attributable risk of anaemia due to *P. falciparum* was 6.3 % (95 % CI 2.5–9.3), 0.9 % (95 % CI 0.4–2.3) for *S. haematobium, and* 12.5 % (95 % CI 9.11–19.52) for stunted malnutrition.

**Conclusion:**

*Plasmodium falciparum*, *S. haematobium*, intestinal helminths and their co-infections were uncommon in our school-age children. Stunting exacerbated the extent to which malaria was associated with loss in Hb concentration.

**Electronic supplementary material:**

The online version of this article (doi:10.1186/s13104-016-2025-3) contains supplementary material, which is available to authorized users.

## Background

The South Tongu District is one of the 25 districts of the Volta region of Ghana drained by the Volta river and its tributaries [1]. In recent times, comprehensive initiatives with health education, personal hygiene instructions, and mass chemotherapy treatment in school-age children and high-risk adults have been emphasized in the Volta river basin [2–4]. There is the schistosomiasis control initiative Ghana (SCIG) which aims to implement an integrated national plan for sustainable control of schistosomiasis in Ghana [5]. There is also the West African international parasite control (WACIPAC) whose initiatives have included the institution of preventive measures on schistosomiasis and soil transmitted helminths [6]. Malaria is endemic in Ghana and represents a major cause of morbidity and mortality in children of school-going age. An accurate information on burden of malaria at the district level is requisite both to plan local parasitic control efforts and to measure the impact of such efforts especially in riparian communities [7–9]. But targeting the correct set of interventions to these major parasitic groups may not be done effectively without regard for nutritional status [2, 10]. Nutrition plays a major role in maintaining health; and malnutrition appears to generate vulnerability to a wide variety of diseases and general ill health. Whereas animal studies suggest that improved nutritional status is protective against malaria, consensus has yet to be reached regarding its effects in human populations [11]. More recent studies indicate either no evidence of benefit or some benefits resulting from nutritional adequacy [2, 10]. In this paper, we describe as our primary outcome, data from a district survey on school-age children (SAC) (6–13 years) regarding the prevalence of malaria, schistosomiasis, and intestinal helminths in riparian community settings that had hitherto encountered several episodes of preventive chemotherapy including mass deworming exercises; and explore relationships between such infections and nutritional inadequacies.

## Methods

### Study area and population

The study was conducted during the first annual rainy season (April through July) of 2012 in the South-Tongu district, an area with seasonal malaria in Volta region, Ghana [4, 12]. The study area comprises 594.75 km^2^ located on Latitude 5°58′37.2′′ and Longitude 0°38′49.2′′. Rainfall is bimodal with latter rains in September to November [13]. The main river draining the district is the Volta, which runs along its western border, but it is also drained by lakes, streams and lagoons in the southern sector of the district. Preliminary surveillance along the river bodies revealed lacustrine conditions favourable to mosquito breeding, growth of aquatic weeds and breeding of snails. Malaria, schistosomiasis, soil-transmitted helminths and onchocerciasis control programmes are active in this area [14, 15]. The district has the Comboni hospital which offers primary health care and diagnostic services. According to hospital records, malaria remains the number one cause of hospital admissions and child morbidity and mortality in the district, with the predominant parasite being *Plasmodium falciparum*. Malaria transmission occurs throughout the year with peaks during the two rainy seasons.

### Study design

**Fig. 1:**
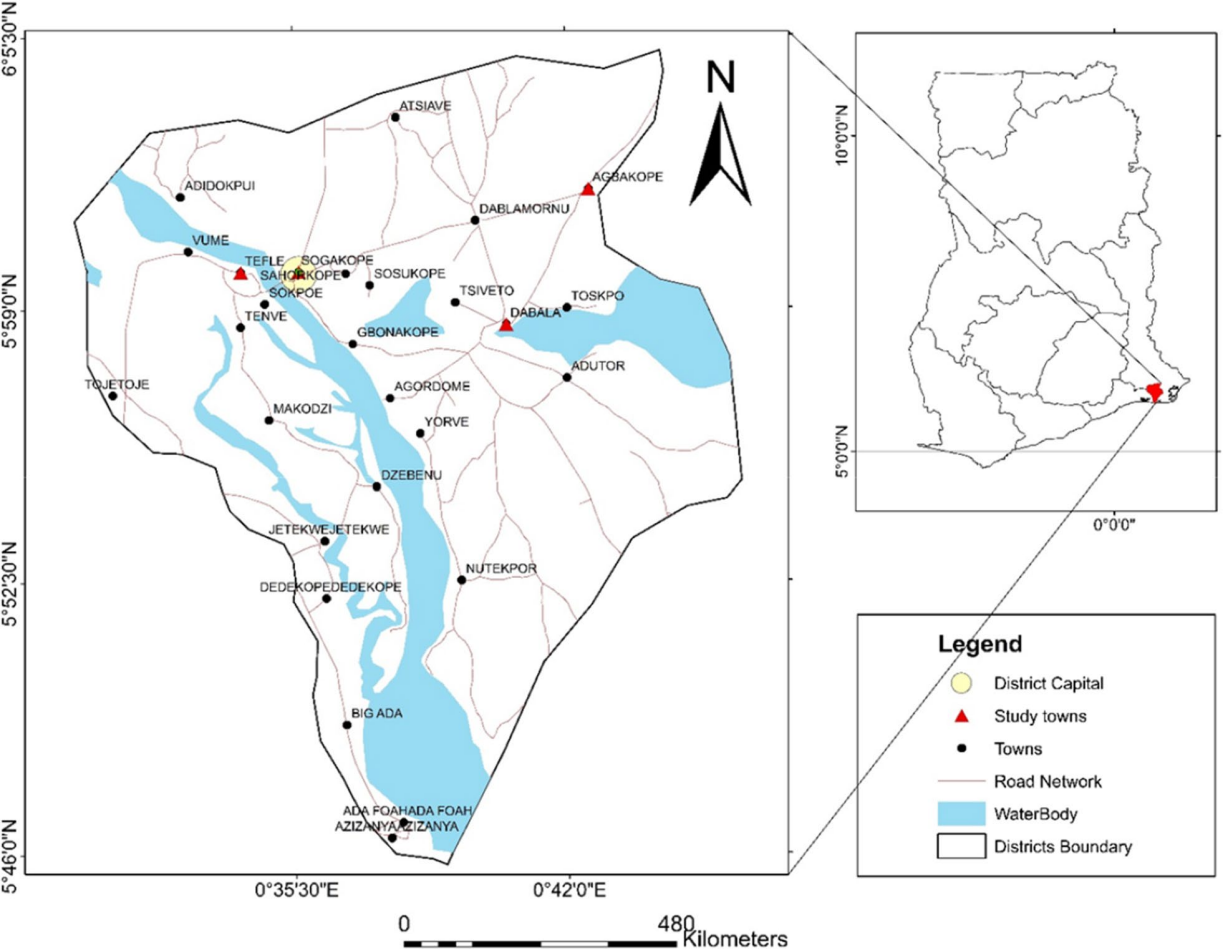
Map of Ghana showing South-Tongu district (Study sites indicated with *arrows*). Tefle and Sogokope are to the eastern border of the district. Dabala and Agbakope occupy the western border of South-Tongu. All study regions are riparian communities

The target population comprised school-age children (ages 6–13 years) without manifestations compatible with malaria in the past 14 days. Children on nutritional supplements were excluded. Four primary schools across the district namely Dabala Evangelical, Tefle Presbyterian Church of Ghana, Sogakope Cuniberto, and the Tsavanya district assembly basic in Agbakope were selected (Fig. 1). Children were selected using a 2-stage cluster sampling procedure. At the first sampling stage, four of seven district circuits in the study area were sampled systematically, with a sampling probability proportional to size, based on Ghana statistical service census data of 2009, as measured by the number of primary schools. At the second sampling stage, one primary school was randomly selected within each circuit. Since malaria and helminth infections detected by blood, urine and stool analysis were our focus of interest, this study was confined to children who could provide all study samples.

### Data collection

Fieldwork took place from April to June of 2012. Parents and guardians were requested for assistance in provision of information. Interviews were conducted in the local dialect, Ewe, spoken by study participants. Data were abstracted through interviews using standardized questionnaires with the help of trained field workers (Additional file 1). Data was collected regarding the following: demographics (gender and age), malnutrition status (stunted, underweight, wasted), child’s knowledge of causes of malaria, helminth infections and schistosomiasis, deworming, availability of toileting facilities in household, sources of household water, child’s activity in any river and parent/guardian educational level. We also determined the household socio-economic status using proxy measures based on the World Bank asset scores for Ghana [16]. For each enrolee, anthropometric measurements including height and weight were determined. The anthropometric indices height-for-age (HA), weight-for-age (WA), weight-for-height (WH) were expressed as Z-scores using the WHO child growth standards [17, 18]. For all children, axillary temperature was measured, and inquiry was made if they had experienced fever in the previous 14 days.

### Sample collection and examinations

Children were provided with plastic containers and requested to bring about 3 g of stool and 20 ml of urine at 10:00 a.m. in the morning. Stool samples were processed within 6 h of collection and examined microscopically within 1 h of preparation. They were examined for *Schistosoma mansoni* and intestinal helminths by wet mount for ova and larvae. The formol-ether concentration technique was used to confirm the presence of parasites in stool. Intensity of infection was assessed by egg count and expressed as mean eggs per gram of faeces (EPG). Ten millilitres of urine samples were examined for *S. haematobium* eggs using the nucleopore filtration method. The intensity of *S. haematobium* infections was calculated as the number of ova per 10 ml of urine. For haemoglobin (Hb) measurements and malaria testing, approximately 3 ml of blood samples were collected from each pupil. Thick blood smears were prepared, stained with Giemsa and examined microscopically for detection, identification and quantification of malaria parasites. Parasite densities were determined with absolute white blood cells (WBC) counts as a ratio of *P. falciparum* counts relative to 200 WBC in thick films. Hb concentrations were determined using the cyanmethaemoglobin method. Briefly, blood aliquots of 0.02mls were put into five milliliters of Drabkin’s solution to make a 1 in 250 dilution. The solutions were well mixed and incubated in the dark for 10 min before Hb estimations (g/dl) in a colorimeter at 540 nm wavelength. Quality control was performed by randomly selecting and microscopically re-examining 10 % of blood, urine and stool preparations by an experienced independent technician blinded to all previous results.

### Data analyses

Data from interviews and parasitological investigations were captured into Microsoft Excel database, and exported into Statistical Package for Social Sciences (SPSS, version 16) for editing and statistical analyses. Missing data were excluded from analysis. Any anaemia was defined as Hb <12 g/dl; severe anaemia, Hb <7 g/dl; moderate anaemia, Hb 7 to <10 g/dl; and mild anaemia, Hb 10–11 g/dl. Based on positive samples only, infection intensities were calculated as parasites per microlitre of blood for *P. falciparum*, EPG of faeces for intestinal helminths, and eggs per 10 ml of urine for *S. haematobium*. Children were classified as stunted, underweight or wasted when their HA, WA or WH Z-scores were <−2 below the reference mean, respectively. A child was identified as being malnourished with a score of <−2 in 1 of the HA, WA, or WH assessment [17, 18]. Point estimates of statistical significance are indicated with two tailed *P* < 0.05. The proportion of anaemia cases attributable to malnutrition or to specific parasitic infections was estimated as population attributable risk percentages (PAR%) [19, 20]. Descriptive statistics (frequencies and cross-tabulations) were used to determine the prevalence of parasitic infections. For continuous variables, standard weighted-mean statistics using Student’s t test or one-way analysis of variance (ANOVA) were used to respectively estimate differences in two or more population means. Categorical data were compared across study parameters using Chi square or the Fisher’s exact test where appropriate. The Mantel–Haenszel Chi square was used to test for trends in linearity. Correlations were assessed, where appropriate, with Pearson coefficient (r) or Spearman’s rho (r_s_) and their coefficient of determination (r^2^ or r_s_^2^). Univariate comparisons between study outcomes and covariates were computed with Chi square tests and unadjusted odds ratios (OR) at 95 % confidence interval (CI). From univariate analyses, variable with a *P* < 0.05 were analyzed in multivariate logistic regression models to identify independent risk factors. Predictive accuracy of the models was assessed by Hosmer and Lemeshow goodness-of-fit test with *P* > 0.05 indicating that the model predicts accurately on average. The area under the receiver operating characteristic (ROC) curve >0.7 was used to evaluate the discriminatory capability of models. Multiple linear regression was used to compare stunted and non-stunted children regarding their associations between malaria, hemoglobin concentration, anaemia, *P. falciparum* parasitaemia, and *S. haematobium* egg count. Effect size determination and possible interaction of the variables were computed with analysis of covariance (ANCOVA).

## Results

Overall 591 SAC were available for sampling based on parents/guardians informed consent. One-hundred and eighty seven of these were excluded from the study. Reasons for exclusion included absence from school (n = 51), unwillingness to provide blood, urine and stool samples (n = 46) and the refusal of children to assent (n = 74). A total of 404 children including 198 girls and 206 boys in the south-Tongu district of Ghana were enrolled. The mean age of SAC was 8.2 years (range 6–13 years); and about 94 % had received antihelminthic drugs in the previous 3 months.

### Prevalence of infection, malnutrition and anaemia

Table 1 summarizes the baseline characteristics of our study population. Of the total 404 children, the prevalence of asymptomatic malaria was 9.2 % (n = 37/404; 95 % CI 6.6–12.5)—*P. falciparum* accounted for all infections. Ten children (2.5 %; 95 % CI 1.2–5.4) harboured *S. haematobium* ova. None of the children was identified with intestinal helminths nor suffered from co-infections with multiple species or different parasites. About 22 % of the children were stunted with a mean HA Z score of −1.90 ± 1.23; whilst 21.5 % were underweight with a mean WH Z score of −0.88 ± 0.57. The prevalence of wasted children was 8.4 % with a mean WA Z score of −2.04 ± 0.9. About 295 (73.0 %) of the children suffered from anaemia—5.7 % (n = 17) of which were severe.

**Table 1:** Baseline study outcomes

Parameter	Description	Number of children	
		Examined	Results
Malaria	Prevalence, 9.2 %	404	37
Causative species	*Plasmodium falciparum only*	404	37
Children with asymptomatic *P. falciparum* infections	Asexual *parasitaemia,* no clinical symptoms	47	37
Children with severe malaria	Asexual *parasitaemia,* severe disease symptoms	47	0
Children with uncomplicated malaria	Asexual *parasitaemia*, history of fever, absence of severe disease symptoms	47	0
Intestinal helminths	None identified	404	0
Urinary schistosomiasis	Prevalence, 2.5 %	404	10
Causative species	*Schistosoma haematobium only*	404	10
Haematuria as prognosis for *S. haematobium* infection	None identified	404	0
Co-infections with different parasites	None identified	404	0
Anaemia of any type	73.0 %	404	295
Severe	4.2 %	404	17
Moderate	47.3 %	404	191
Mild	21.5 %	404	87
Weight-for-age Z score <−2 (stunted)	22.3 % (95 % CI 15.0–35.4)	404	90
Height-for-age Z score <−2 (wasted)	8.4 % (95 % CI 4.6–13.8)	404	34
Weight-for-height Z score <−2 (underweight)	21.5 % (95 % CI 17.7–25.9)	404	87

Height-for-age Z score, weight-for-height Z score, and weight-for-age Z score expressed with standard deviation; *CI* confidence interval

### Intensity of infections

Among *P. falciparum* infected children, the mean parasitaemia was 4641 parasites/µl (95  % CI 1812–8973) of blood. About 24.3 % (n = 9) of the children had low *P. falciparum* parasitaemia <500 parasites/µl of blood and more than half (n = 22; 62 %) had moderate parasitaemia ranging between 500 and 10,000 parasites/µl of blood. A few participants (n = 5/37, 13.5 %) had high parasite count >10,000 parasites/µl of blood. Of the ten infected by *S. haematobium*, six had light infections (<50 ova per 10 ml urine) with a mean of 37 ± 12. Four children suffered heavy *S. haematobium* infections (>50 ova per 10 ml urine) with egg intensity of 103 ± 21. The mean ova count for *S. haematobium* infections was 63 ± 24.

### Univariate and multivariate analysis

From Table 2, five factors were identified to be associated with *P. falciparum* infection: older age (11–13 years), being malnourished and stunted, use of repellents as protection against malaria, use of river as source of household water, child’s activity in river, travel in past 4 weeks, and high parent/guardian education. The same variables were significantly associated with *S. haematobium* infection, with the exception of recent travel. However, no anti-helminthic drug use in past 3 months was also significantly associated with *S. haemtobium* infection.

**Table 2:** Characteristics of children in the South Tongu district and bivariate associations with *P. falciparum* and *Schistosoma haematobium* infections

Variables (number)	*P. falciparum* infection		OR (95 % CI)	P value	*S. haematobium* infection		OR (95 % CI)	P value
	Yes (37)	No (367)			Yes (10)	No (394)		
Male gender (n = 206)	20	186	1.2 (0.6–2.3)	0.698	6	200	1.5 (0.4–5.2)	0.75
Age (±1SD)	9.2 ± 2.1	11.4 ± 1.9	–	0.001	10.1 ± 0.9	9.4 ± 1.3	–	0.09
Age group								
5–7 (n = 108)	7	101	0.6 (0.3–1.4)	0.35	6	102	4.3 (1.18–15.5)	0.03
8–10 (n = 130)	7	123	0.5 (0.2–1.1)	0.07	2	128	0.5 (0.1–2.5)	0.50
11–13 (n = 166)	23	143	2.5 (1.2–5.1)	0.006	2	164	4.3 (1.2–15.5)	0.03
Malnourished (n = 211)	25	186	2.0 (0.9–4.5)	0.05	5	206	0.9 (0.3–3.2)	1
Stunted (n = 90)	16	74	3.0 (1.5–6.07)	<0.001	2	88	0.9 (0.2–4.2)	1
Underweight (n = 87)	5	82	0.3 (0.1–0.9)	0.02	1	86	0.4 (0.1–3.2)	0.50
Wasted (n = 34)	4	30	1.36 (0.5–4.1)	0.76	2	32	2.82 (0.7–13.8)	0.20
Haemoglobin (±SD)	9.1 ± 1.1	13.5 ± 2.4	–	<0.001	11.1 ± 0.5	11.7 ± 0.8	–	0.116
Any anaemia (n = 295)	33	262	3.31 (1.1–9.6)	0.006	5	290	0.4 (0.1–1.3)	0.14
Severe (n = 17)	4	13	3.30 (1.0–10.7)	0.06	2	15	5.5 (1.1–28.1)	0.07
Moderate (n = 191)	24	167	2.49 (1.2–5.1)	0.009	2	189	0.3 (0.1–1.3)	0.11
Mild (n = 87)	5	82	0.54 (0.2–1.4)	0.21	1	86	0.4 (0.04–3.2)	0.47
Protection against malaria^a^ (307)	23	284	0.48 (0.2–1.0)	0.038	3	304	0.12 (0.03–0.50)	0.002
Bed nets (131)	2	129	0.07 (0.02–0.3)	<0.001	0	131	–	
Repellents (13)	5	8	7.0 (2.1–22.7)	<0.001	2	11	8.7 (1.6–45.8)	0.04
House screens (70)	5	65	0.7 (0.3–1.9)	0.522	1	69	0.5 (0.07–4.2)	0.70
Medication in past 4 weeks (93)	11	82	1.5 (0.7–3.1)	0.310	0	93	–	–
Childs knowledge of cause of malaria								
Yes (161)	19	142	1.9 (0.9–3.8)	0.1	2	159	0.37 (0.07–1.76)	0.32
No (243)	16	227			8	235		
Reported travel outside home in 4 weeks								
Yes (79)	16	63	3.6 (1.8–7.4)	0.001	1	78	0.4 (0.05–3.6)	0.69
No (325)	21	304			9	316		
Sources of water								
River (46)	17	29	9.9 (4.6–20.9)	<0.001	5	41	8.6 (2.4–31.0)	0.002
Borehole (283)	10	273	0.1 (0.06–0.3)	<0.001	2	281	0.1 (0.02–0.510	0.01
Tap water (75)	10	65	1.7 (0.8–3.7)	0.16	3	72	1.9 (0.5–7.5)	0.4
Activity in river								
Yes (90)	22	68	6.4 (3.2–13.1)	<0.001	6	84	5.5 (1.5–20.1)	0.001
No (314)	15	299	–		4	310		
Frequency of activities per week in river	10.3 ± 2.4	10.1 ± 1.1	–	0.36	11.3 ± 1.4	9.2 ± 2.0	–	0.001
Toilet facilities								
Yes (220)	22	198	1.3 (0.6–2.5)	0.5	5	215	0.8 (0.2–2.9)	1.0
No (184)	15	169	–		5	179		
Child’s knowledge on schistosomiasis								
Yes (9)	2	7	2.9 (0.5–14)	0.19	1	8	5.3 (0.6–47.4)	0.2
No (395)	35	360	–		9	386		
Caregivers knowledge on helminths infections								
Yes (48)	4	44	0.9 (0.3–2.6)	0.1	2	46	1.9 (0.4–9.2)	0.6
No (356)	33	323	–		8	348		
Parent/guardian education								
Primary (121)	10	111	0.8 (0.4–1.8)	0.68	2	119	0.6 (0.1–2.8)	0.7
Secondary (101)	11	90	1.3 (0.6–2.7)	0.4	3	98	0.4 (0.1–1.67)	0.3
Tertiary (14)	4	10	4.3 (1.2–14.5)	0.03	1	13	3.3 (0.3–27.6)	0.3
None (168)	12	156	0.6 (0.3–1.33)	0.24	3	165	0.6 (0.15–2.3)	0.56)
Socioeconomic score of household								
Lowest (388)	31	357	0.1 (0.05–0.4)	<0.001	7	381	0.08 (0.02–0.34)	0.005
Middle (13)	5	8	7.1 (2.2–22.6)	0.003	3	10	16.5 (3.7–73.6)	0.002
Highest (3)	1	2	5.1 (0.5–57.2)	0.25	0	3	–	–
Use of anti-helminth drugs in past 3 months								
Yes (378)	23	355	0.2 (0.09–0.60)	0.005	7	371	0.08 (0.02–0.33)	0.002
No (26)	14	12			4	22	–	–

^a^Number of children engaging each activity, overlap due to multiple responses; None of *P. falciparum* infected children reported with fever in past 48 h and/or current axillary temperature ≥ 37.5 °C; Continuous variables reported as geometric means with standard deviations (SD); *OR* odds ratio, *CI* confidence interval

In adjusted models (Table 3), older age 11–13 years [adjusted odds ratio (AOR), 3.1; 95 % CI 1.1–5.6] and stunted malnutrition (AOR, 8.72; 95 % CI 4.8–25.1) were identified as independent risk factors *P. falciparum* infection. Also, a unit increase in Hb concentration was determined to be putatively protective against *P. falciparum* infection (AOR = 0.71; 95 % CI 0.42–2.3). Stunted malnutrition was the strongest predictor of *S. haematobium* infection (AOR = 11; 95 % CI 3.1–33.6). The frequency of a child’s activities per week in river was identified as an independent risk factor (AOR = 1.68 per one activity increase) for *S. haematobium* infection—with infected children engaging in river activities almost twice as frequent as those uninfected. Reported use of anti-helminthic drug in the past 3 months was found to be 87 % protective against *S. haematobium* infection.

**Table 3:** Multivariate adjusted associations for predictors of *P. falciparum* and *Schistosoma haematobium* infections

Attributable risks	Level	Adjusted OR	95 % CI	P value
Model I: *P. falciparum* *infections*				
Child age 11–13 years	2 years increase	3.1	1.1–5.6	0.01
Stunted malnutrition	Yes/no	8.72	4.8–25.11	0.008
Haemoglobin concentration	1 unit increase	0.71	0.42–2.3	0.002
Model 11: *S. haematobium* infections				
Stunted malnutrition	Yes/no	11.6	3.1–33.6	0.001
Reported use of anti-helminthes drugs in past 3 months	Yes/no	0.13	0.08–1.31	0.002
Number of activities per day in River	1 activity increase	1.68	0.84–3.61	0.01

*OR* odds ratio, *CI* confidence interval

### Trends in *P. falciparum* infections

**Fig. 2:**
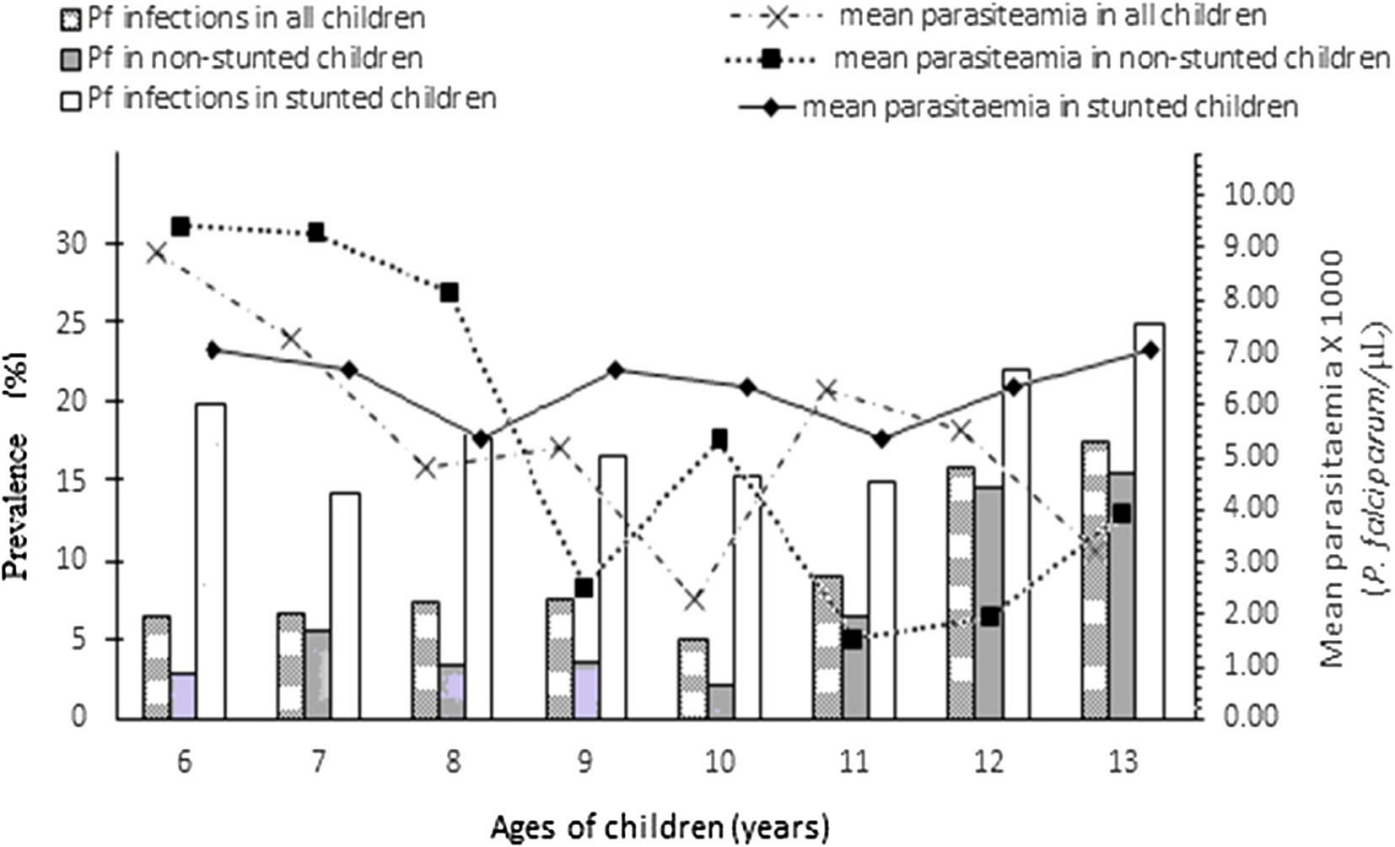
Age-specific distribution of *P. falciparum* infections in children with stunted and non-stunted malnutrition compared to mean parasitaemia. Pf, *Plasmodium falciparum.* For *P. falciparum* infections among all children, the trend of infections declines with increasing age (X^2^ for trend, *P* = 0.036); among non-stunted children, the trend of infections declines with increasing age (X^2^ for trend, *P* = 0.04); among stunted children, a zero linear trend observed with increasing age (X^2^ for trend, *P* = 0.196). For mean parasitaemia among all children, non-significant negative correlation with age (r = −0.650, r^2^ = 0.423, *P* = 0.081); among non-stunted children, significant drift towards lower parasitaemia of *P. falciparum* with increasing age (r = −0.816, r^2^ = 0.666, *P* = 0.014); among stunted children, *P. falciparum parasitaemia* relatively remained similar with increasing age (r = −0.087, *P* = 0.837)

**Fig. 3:**
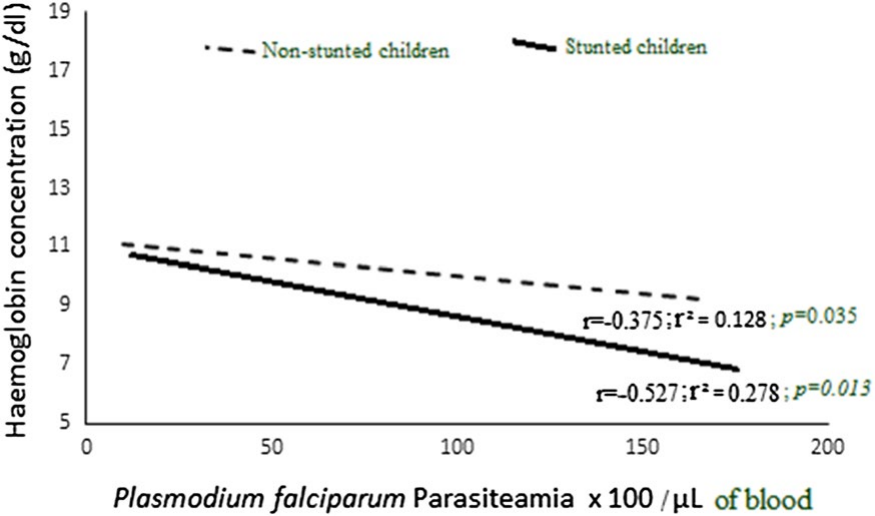
Haemoglobin versus *P. falciparum* parasitaemia in stunted and non-stunted children. Within the stunted cohort a significant correlation between haemoglobin concentration and parasite density (r = −0.527; r^2^ = 0.278; *P* = 0.013) was observed. A similar association was present in the non-stunted group albeit at a slower rate (r = −0.375; r^2^ = 0.128; *P* = 0.035)

The associations between significant terms in the multivariate regression analysis for *P. falciparum* infections were further assessed to test the significance of the product terms directly i.e., age, Hb concentration and stunted malnutrition. Figure 2 shows the age-specific distribution of *P. falciparum* infections among all study children—the trend of infections declined with increasing age (X^2^ for trend, P = 0.036). When data was adjusted for stunting, the frequency of infections showed no tendency to increase or decrease with age (X^2^ for trend, P = 0.196). Meanwhile, a significant drift towards lower parasitaemia with increasing age was observed in non-stunted children (r = −0.816, r^2^ = 0.666, *P* = 0.014) (Fig. 2). The mean parasite density of *P. falciparum* also remained relatively unchanged with increasing age (r = −0.870, *P* = 0.837). Among the infected children, the stunted group showed higher (*P* < 0.001) mean parasitaemia level (3218 ± 1184 parasites/µl) than their non-stunted counterparts (2064 ± 1287 parasites/µl). We observed within the stunted cohort a significant correlation between Hb concentration and parasite density (r = −0.527; r^2^ = 0.278; *P* = 0.013); such association was also present in the non-stunted cohort albeit at a slower rate (r = −0.375; r^2^ = 0.128; *P* = 0.035) (Fig. 3).

### Associated effects of *P. falciparum*, Hb concentration and stunting

**Fig. 4:**
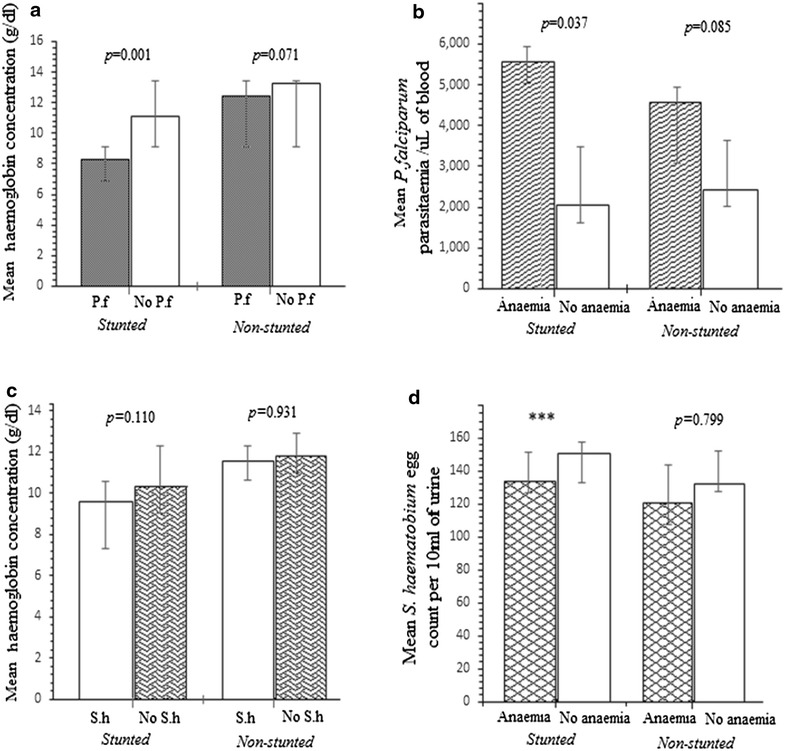
Comparisons of stunted and non-stunted children regarding the associated effect of **a** *Plasmodium falciparum* (P.f) on mean haemoglobin concentration. The *P. falciparum*-associated decrease in mean haemoglobin concentration was significant in stunted children; **b** anaemia on mean *P*. *falciparum* parasitaemia. Among stunted and non-stunted children, the anaemia-associated decrease in mean parasitaemia were respectively non-significant; **c** *Schistosoma haematobium* (S.h) on mean haemoglobin concentration. The *S. haematobium*-associated decrease in mean haemoglobin concentration was not significant in stunted and non-stunted children; **d** anaemia on mean *S. haematobium* infection intensity. There were no significant anaemia-associated increases in *S. haematobium* egg intensity in stunted or non-stunted children

Figure 4a compares stunted and non-stunted children regarding their association between malaria and Hb. The *P. falciparum*-associated decrease in mean Hb concentration was 2.82 g/dl (95 % CI 1.63–4.1 g/dl; *P* = 0.001) in stunted children, and 0.75 g/dl (95 % CI 1.59–0.085 g/dl; *P* = 0.076) in the non-stunted cohort. Of the *P. falciparum* infected children, 93.7 % (n = 15/16) of those stunted were anaemic whiles 90.4 % (n = 19/21) of the group without stunting suffered from anaemia. Among the stunted and non-stunted children, the anaemia-associated decrease in mean parasitaemia were 3500 parasites/µl of blood (95 % CI 262.46–6737.54 parasites/µl of blood; *P* = 0.0358) and 2127 parasites/µl of blood (95 % CI −0.27 to 4.53; *P* = 0.085) respectively (Fig. 4b). The PAR % of anaemia attributable to *P. falciparum* infection was 6.3 % (95 % CI 2.5–9.3), and 12.5 % (95 % CI 9.11–19.52) for stunted malnutrition.

### *S.haematobium* infections and associated effects

The PAR % of anaemia attributable to *S. haematobium* infection was 0.9 % (95 % CI 0.4–2.3). When we compared the associated effect of *S. haematobium* infection and Hb concentration between the two cohorts, *S. haematobium*-associated decrease in mean Hb concentration was 0.6 g/l (95 % CI −158–1.358 g/l; *P* = 0.111) in stunted children; and 1.2 g/l (95 % CI −0.519–2.92 g/l; *P* = 0.170) in those without stunting (Fig. 4c). Similarly, there was no significant anaemia-associated increases in *S. haematobium* egg intensity in stunted or non-stunted children (Fig. 4d). Also no correlation was observed between *S. haematobium* egg intensity and Hb concentration (r = 0.2784; *P* = 0.117).

## Discussion

The study showed low numbers of asymptomatic *P. falciparum* infections with largely low to moderate level parasitaemia. About half of the affected suffered from stunted malnutrition with a high proportion of moderate to severe anaemia. The prevalence of urinary schistosomiasis was 2.5 %, with low to high ova counts for *S*. *haematobium*—the principal manifestations were stunted malnutrition and moderate anaemia. For two reasons, our findings of low malaria levels deserve particular attention. First, *P. falciparum* accounted for all malaria cases reported here, and the level of infection using comparative methodology was lower than that reported for other malaria-affected children in Ghana [12, 21, 22], Tanzania [23] and in many other reviews spanning several endemic regions in Africa [24, 25]. Second, the parasite rates from our study was obtained through primary school survey and represent aggregated rates for communities in each school’s catchment area. Also, similar low levels of *P. falciparum* infections were observed between the various community schools. Taken together, the findings should not be considered as an artefact of a single study period but as results which perhaps points to a temporally stable infection rate among SAC in the South-Tongu district. The results is also in keeping with previous mass deworming, malarial prevention and schistosomiasis control exercises in this region [2–5]. Our data however suggest that despite the improved control efforts, there remain many inhabitants of riparian communities that suffer from urinary schistosomiasis [26]. Although we are not privy to the full repertoire of drugs admitted during these sessions, children had mostly been on praziquantel in various combinations with other drugs including antimalarial.

In multivariate analysis, the reported usage of antihelminthic by study respondents was determined to be protective against urinary schistosomiasis. The study also showed that stunted malnutrition was independently associated with increased odds of *P. falciparum* infections. Stunted malnutrition was also present after assessing data separately for *S. haematobium* infections. Stunting is a manifestation of recent and acute under nutrition. Children who are stunted are thought to have increased predisposition to malaria and other parasitic infection for a variety of reasons, most notably through a reduction in the function of T-lymphocytes, deficiencies in antibody formation, diminished complement formation, and degeneration of thymus and other lymphoid tissues [2, 10, 22, 27]. From our study, an increase of one unit of Hb was protective against *P. falciparum* infection and corresponded to an AOR of 0.71 (95 % CI, 0.42–2.3, P = 0.002). Meanwhile, children with stunted malnutrition showed higher parasitaemia levels that negatively correlated with lower Hb concentration. The PAR % of anaemia attributable to *P. falciparum* remained about twofold lower than that of stunted children who accounted for approximately one-eighth of anaemia cases. The data corroborates with our other results regarding stunted-malnutrition-associated decrease in Hb concentration among malaria affected children (Fig. 4). In the low malaria settings of South-Tongu, stunted malnutrition could be contributing to lower Hb concentrations in *P. falciparum* affected children. It is noteworthy that although these observations with stunting were not apparent in schistosomiasis affected children, a clear evidence that stunting is associated with increased prevalence in malarial infection is beyond the scope of this study.

**Fig. 5:**
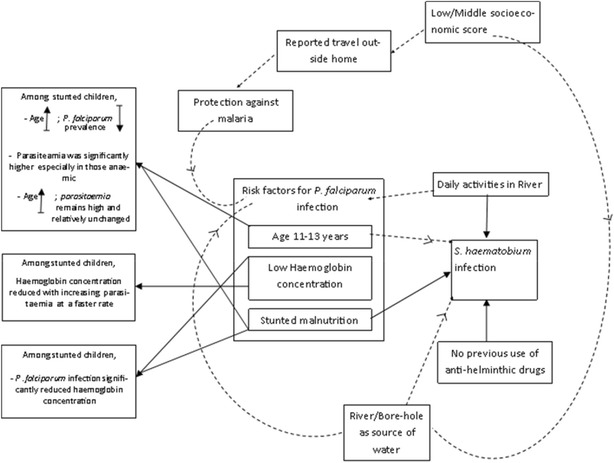
Factors associated with *P. falciparum* and *S. haematobium* infections. *Arrows* indicate risk factor associations; *Dotted arrows* indicate less-definitive relationships; *upward arrow* denotes increase; *downward arrow* denotes decrease; N/C refers to no correlation

It is conceivable that malaria may cause stunting. Yet given that stunting becomes manifest only after a prolonged period of nutritional insufficiencies, and that malaria infection is a transitory state, we consider it more likely that host immunity impairment which results from conditions such as Hb deficiency exacerbate host vulnerability to malaria [2, 22, 27]. A diagram describing the relationship between infections with *P. falciparum, S. haematobium*, Hb concentration and stunted malnutrition is considered in Fig. 5.

There are some potential limitations of this study that must be discussed briefly, and for which our data must be interpreted carefully. First, the studies for helminths used only one stool and urine sample from each child; hence, the proportion of children with low-intensity infections could have been misclassified as uninfected [23, 28]. The prevalence of helminth infections is therefore likely to be underestimated in this study. Second, another issue worth mentioning is the limited sampling size and the concomitant low levels of urinary schistosomiasis and other helminth infections. Whereas this may reflect the relative incidence of organisms in a riparian community with active preventive and curative programmes against parasitic infections, a more large-scale survey is likely to be with little bias for *P. falciparum* and *S. haematobium*. Last, other haematological markers such as serum C-reactive protein and soluble transferrin receptor concentrations were not determined [27]. The effect estimates of such parameters would present a more holistic discussion on our data. Despite the shortcomings, our study demonstrate that malaria, urinary schistosomiasis, soil-transmitted helminths, and co-infections with these parasites are uncommon among children in South-Tongu district; and highlights the success of parasite control programmes in our study area.

## Conclusions

The silver lining to this study is the fact that our data shows malnourishment that cause stunting may also exacerbate the extent to which malaria is associated with deficiency in Hb concentration [2, 10, 22, 27]. We suggest further studies on whether nutrition intervention therapy targeted in stunted children may help reduce the anaemia burden of *P. falciparum* infections.

## Additional file

10.1186/s13104-016-2025-3 Questionnaire for data collection. Data on participants characteristics were collected via interviews using standardized questionnaires. The questionnaire abstracted information regarding the following demographics (gender and age), child’s knowledge of causes of malaria, helminth infections and schistosomiasis, deworming, availability of toileting facilities in household, sources of household water, child’s activity in any river and parent/guardian educational level. We also determined the household socio-economic status using proxy measures based on the World Bank asset scores for Ghana. For each enrolee, anthropometric measurements including height and weight were determined.

## Abbreviations

SAC: school-age children
*S. haematobium*: *Schistosoma haematobium*
*P. falciparum*: *Plasmodium falciparum*
AOR: adjusted odds ratio
CI: confidence interval
SCIG: schistosomiasis control initiative Ghana
WACIPAC: West African international parasite control
HA: height-for-age
WH: weight-for-height
WA: weight-for-age
EPG: eggs per gram
WBC: white blood cells
Hb: haemoglobin
r: Pearson coefficient
r_s_: Spearman’s rho
ROC: receiver operating characteristic
ANCOVA: analysis of covariance
PAR: population attributable risk
