# Supplementary material for: Malaria, helminths and malnutrition: a cross-sectional survey of school children in the South-Tongu district of Ghana
Source: BMC Res Notes. 2016 Apr 27;9:242. doi: 10.1186/s13104-016-2025-3 (PMC4847346; doi:10.1186/s13104-016-2025-3)
Supplement: Supplementary file 1 — 10.1186/s13104-016-2025-3 Questionnaire for data collection. Data on participants characteristics were collected via interviews using standardized questionnaires. The questionnaire abstracted information regarding the following demographics (gender and age), child’s knowledge of causes of malaria, helminth infections and schistosomiasis, deworming, availability of toileting facilities in household, sources of household water, child’s activity in any river and parent/guardian educational level. We also determined the household socio-economic status using proxy measures based on the World Bank asset scores for Ghana. For each enrolee, anthropometric measurements including height and weight were determined. [file 13104_2016_2025_MOESM1_ESM.pdf]

Malaria, helminths and malnutrition in school children in South-Tongu district of Ghana

Participant Data collection Instrument

A) ***For data collecting team only***

Assign Pupil ID…………………………………………Age……Sex…...

Assign School ID…………………………………………………………

Assign Town ID …………………………………………………….

B) ***For children participants***

1. Do you know something about worms? Yes……..... No……………
2. Do you know how a child gets worm infection? Yes……… No….……
3. Does your child goes to fetch water in the river? Yes………… No……..………
4. What is the source of your drinking water? Tap water…… Bore hole….…River……
5. Do you have toilet facility in the house? Yes…………No…………………
6. If no how far is the facility from your home?...
7. How do you dispose of your refuse?...
8. How far is the refuse dump from your house?..
9. What is the playing pattern of your?..
10. Do you tell your child to wash his/her hands before eating? Yes….No….
11. Is there any stagnant water bodies in or around this community? Yes…No....
12. How is your family protected from mosquito bites at night? The use of

    Mosquito nets………Mosquito coils……Sprays………Others……………
13. Weight…………………………….. Height
14. Was blood sample? Yes…………No…………….

Have you heard about malaria? Yes…………No……………

15. Do you know about malaria ………………………………

16. What causes malaria? Mosquitoes………..Houseflies…………..Bad food………… Others (specify)………………….

17. Have you travelled outside this town in past 4 weeks? Yes………..No……..

18. Do you fetch water for the home? Yes…………No…………

19. Where do you fetch water from?

River…………….Bore hole…………………Tap water…………………

20. Do you bath or wash or play in any of the rivers in the town? Yes……No….

21. How many times in a week do you bath or wash or play in the river…………

22. How far is your house from nearest river (measure distance in Km)…………

Do you have a toilet facility in your house? Yes……No………

23. Have you heard of *Schistosomiasis*? Yes………..No………

24. What causes schistosomiasis? Mosquitoes………Contact with the river……..Flies……………Others (specify)

25. Have you been part of a mass deworming exercise? Yes…….No………

26. When were you involved in the exercise in question 24?.................................

27. In question 24, what drugs did you receive?

....

....

28. Any other information

………………………………………………………………………………………

………………………………………………………………………………………

………………………………………………..

....

....

....

....

....

C) ***For either parents or guardians***

29. Have you heard of *Schistosomiasis*?   Yes………..No……….

30. What causes schistosomiasis? Mosquitoes………Contact with the river……..Flies……………Others (specify)

31. Do you know of helminthes ? (let respondents explain the knowledge)

………………………………………………………………………………………

………………………………………………………………………………………

………………………………………………………………………………………

………………………………………………………………………………………

………………………………………………………………………………………

……………………………………………………………………………

32. Education status (Indicate caregiver type)?

Primary……….Secondary……….Tertiary…………None

33. Occupation of caregiver…………………………………

34. List properties in the house

……………………………………………………………………………………

……………………………………………………………………………………

……………………………………………………………………………………

……………………………………………………………………………………

……………………………………………………………………………………

……………………………………………………………………………………

……………………………………………………………………………………

……………………………………………………………………………………..

34. Any other information.

……………………………………………………………………………………

……………………………………………………………………………………

……………………………………………………………………………………

……………………………………………………………………………………

……………………………………………………………………………………

……………………………………………………………………………………..

Thank you
